# Correlation between hand functioning, cognition and quality of life of CP children: 2-year prospective randomized study

**DOI:** 10.1192/j.eurpsy.2021.248

**Published:** 2021-08-13

**Authors:** T. Voloshyn, V. Kozyavkin

**Affiliations:** 1 Neurology And Medcial Psychology, KIRC, Truskavets, Ukraine; 2 Neurology, KIRC, Truskavets, Ukraine

## Abstract

**Introduction:**

For treatment of CP patients multidisciplinary approach is often promoted. But it is not cost-efficient to involve all medical staff and variety of diagnostic/intervention tools for every patient.

**Objectives:**

Having knowledge on pivotal manifestations of CP and what are they related to would ease multidirectional approach in practice. It will shorten a search of pathologies only to those related to main manifestations related to quality of life.

**Methods:**

611 children aged 2 to 18 years (mean age–6y5m) with CP(G80.0-G80.9): examined by 3 independent doctors. 56%males, 44%females. Randomized blinded assessment. Fine hand function assessed by “9-hole peg”,“Box and Blocks” tests, dynamometry. Intellectual functioning assessment was done using Raven Matrices. Quality of Life(QoL) assessment according to Caregiver Priorities and Child Health Index of Life with Disabilities (CPCHILD). The intraclass correlation coefficient(ICC) was used for finding out discrepancies between observers. Inferencial statistics including 95%CI and P-value.

**Results:**

ICC coefficient between observers was highly reliable–0.93(95%CI:0.89–0.95). The mean QoL score for children GMFCS levels I and II was 58.5(SD 16.6), for GMFCS III, IV and V children–22.4(SD13.4). QoL was more related to fine hand functioning(r=0,344) than to cognition(r=0,295). There was a strong correlation bond between fine hand functioning and cognition (r=0,663). In case fine hand function improved positive changes in cognition were observed in 74% of subjects(p<0,05). Correlation between grasp power and IQ was weak(r=0,184). Grasp power improvement was slightly related to QoL(r=0,102).

**Conclusions:**

Fine hand functioning improves QoL even more that cognition.So training of fine motor skills should be given a priority in case of limited rehabilitation resources.

**Disclosure:**

No significant relationships.

